# Large language models accurately identify decision reasons in verbal reports

**DOI:** 10.1073/pnas.2526798123

**Published:** 2026-06-30

**Authors:** Kamil Fuławka, Ralph Hertwig, Dirk U. Wulff

**Affiliations:** ^a^Center Synergy of Systems, Dresden University of Technology, Dresden 01069, Germany; ^b^ Center for Adaptive Rationality, Max Planck Institute for Human Development, Berlin 14195, Germany; ^c^ Faculty of Psychology, University of Basel, Basel 4055, Switzerland

**Keywords:** verbal reports, large language models, decision making, risky choice, prospect theory

## Abstract

Understanding the reasons behind human choices under risk is a central goal of decision scientists, but traditional methods relying on behavioral data are limited by strict invariance assumptions. We introduce a scalable analytical framework using large language models (LLMs) to analyze verbal reports and identify articulated reasons for choice between monetary lotteries. A validated LLM accurately identified predefined decision reasons in participants’ free-text reports, aligning with their actual choices in 95% of trials. Our analysis reveals that the reasons behind people’s decisions vary systematically and are driven more by the structure of the choice problem than by individual differences. Crucially, reasons identified from verbal reports yield more parsimonious and informative representations of decision processes compared to those inferred from choices alone; furthermore, problem-specific reason profiles achieve out-of-sample prediction accuracy that is competitive with established computational models. This work demonstrates that verbal reports are a rich data source and our analytical framework can unlock their potential, delivering results that challenge the field’s foundational invariance assumptions and pave the way for more context-sensitive and interpretable models of human decision making.

## Significance

Understanding why people make the choices they do is central to decision science. We show that large language models can uncover people’s stated reasons from free-text reports, achieving 95% alignment between actual choices and those implied by the identified reasons. Based on this scalable classification approach of decision reasons, we find that the reasons vary primarily with the structure of choice problems, and less so across individuals. Notably, reasons identified from verbal reports provide a more parsimonious account of decision processes than reasons inferred from choices alone, demonstrating the epistemic value of verbal reports. Our findings challenge the field’s reliance on contextual invariance assumptions and establish LLMs as a transformative tool for building more context-sensitive models of human decision making.

Large language models (LLMs) are powerful machine learning systems trained to produce and interpret language with human-like proficiency. Emerging research demonstrates the potential of LLMs to revolutionize studies in behavioral and social sciences—for example, by enabling detailed analysis of text data (1–6). Here, we demonstrate that LLMs can be used to identify the reasons people give when making risky choices; that is, when choosing between monetary lotteries with fully described outcomes and associated probabilities.

Decision scientists have long sought to understand the processes underlying risky choice. This pursuit has yielded numerous models, including the time-honored prospect theory (7, 8) and newer, more predictive models such as BEAST (9). However, these models typically coexist in parallel, offering explanations that are only loosely related—or often entirely disconnected—from one another. For instance, prospect theory and similar models suggest that people select an option based on its subjectively transformed expected utility—a calculation that weighs the subjective values of potential outcomes, each adjusted by their actual likelihood or subjective variants thereof (e.g., subjective probabilities, decision weights). Alternative proposals, including heuristics and other cognitive models, suggest instead that decision makers choose, for example, the option with the best single outcome (maximum gain) (10), that they anticipate regret or disappointment (11), or that they mentally sample potential outcomes (12). Currently, no model fully accounts for the qualitative and quantitative aspects of human decision making (13). Consequently, the process by which people make decisions under risk is imperfectly understood, limiting the ability to accurately anticipate their actions.

This fragmented understanding is at least partly attributable to the field’s traditional emphasis on “hard” behavioral data. Adopting the traditional assumption of economists that talk is cheap, decision scientists have largely subscribed to the revealed-preference approach (14, 15), inferring decision-making reasons solely from choice data. Although this approach has driven considerable progress, it is highly data-inefficient. A large number of choices are usually needed to infer the data-generating process that underlies them (13), but only a limited number can be generated without negatively affecting the validity of behavioral studies (16). In response, researchers have begun incorporating process-tracing tools, such as mouse- or eye-tracking (17–19), that can generate additional quantitative data on prechoice cognitive steps. While these tools have provided useful insights (20–22), they have not fully elucidated how and why people make risky choices, as recent machine learning approaches continue to reveal systematic variation unexplained by existing models (23, 24).

We argue that the limitations of the behavioral-data-only approach arise from unfounded assumptions of invariance (25). To draw inferences from behavioral data, researchers must make repeated observations and assume that these reflect an identical underlying generating process or processes across individuals and situations. Many investigations effectively assume a single process for all individuals in all situations, allowing, at best, only minimal variation through hierarchical distributions and thereby overlooking more substantial and meaningful differences. This contrasts starkly with research that shows variations in decision processes and reasons between people of different ages (26, 27), genders (28), or vaccination attitudes (29), and between situations with varying time pressures (30, 31), affective content (32, 33), ecological outcome distributions (12), or learning modes (34, 35). In particular, many of these findings involved relaxing some assumed invariances while keeping others constant to ensure reliable inference. As a result, risky choice accounts have largely ignored the possibility of radical differences in decision processes or reasons between individuals, situations, and their unique combinations. However, we suspect that these profound variations are widespread and crucial to fully accounting for and anticipating risky human choices.

To overcome the limitations of the behavioral-data-only approach, we propose an additional data source: verbal reports. In verbal reports, individuals articulate their deliberation process during the process or after making a decision. Because this method can provide granular information about how and why individual choices are made, without imposing invariance assumptions across those choices, verbal reports have been highlighted as a promising avenue to uncover the processes of decision-making (36–38). However, systematic analysis of natural language data has proved challenging, leaving its potential largely untapped.

In this study, we develop an analytic framework that begins with a collection of decision reasons as components of decision processes (inspired by current choice theories), then uses LLMs to scalably identify these reasons in people’s verbal reports. We define decision reasons as a minimal implementation of an established concept in decision science that can be implemented both formally and verbally and distinguishes between choice options. For instance, the most probable outcome reason specifies a preference for the choice option whose most likely outcome is more attractive than that of the other option’s most likely outcome. Note that we assume, throughout, lotteries with two options; in principle, our approach can be generalized to lotteries with more than two options and to other decision situations. By estimating the distribution of decision reasons across individuals and situations, we aim to uncover signals of people’s decision processes, including unobserved variability, and use them to predict people’s choices.

**Fig. 1.**
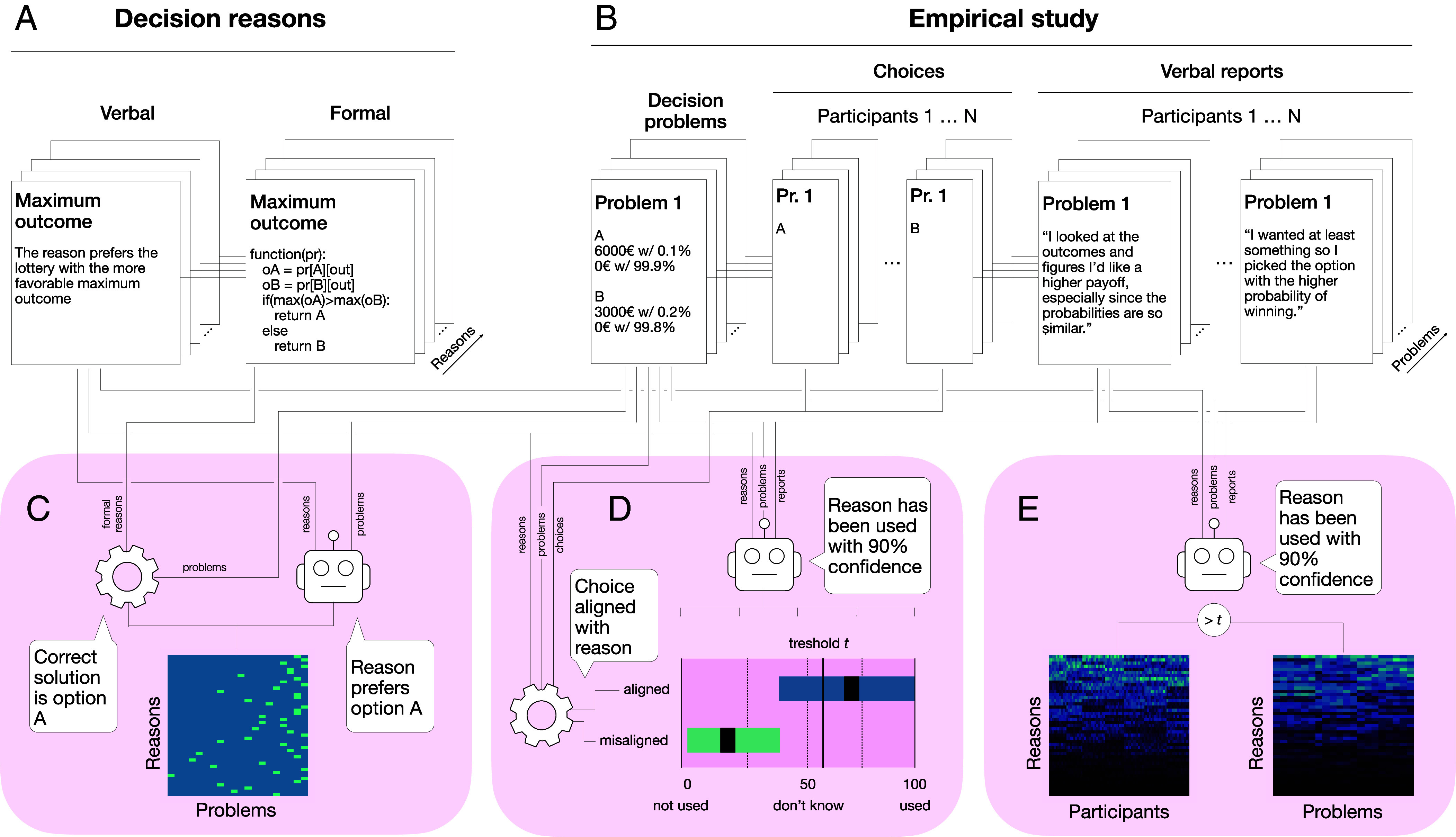
Study pipeline for identifying decision reasons from verbal reports using large language models. (*A*) Decision reasons: the study used a predefined set of 47 decision reasons, each represented in two ways. A verbal representation describes the relevant information and how it is used to make a decision, and a formal representation provides a function that determines the preferred lottery when applied to a choice problem. (*B*) Empirical study: human participants (N=86) completed a behavioral experiment involving 20 monetary lottery problems. For each problem, participants made an incentivized choice and provided a free-text report explaining how and why they made their choice. (*C*) LLMs accurately understand decision reasons and problems: in a validation step, various LLMs were presented with the verbal representations of decision reasons from (*A*) and choice problems from (*B*), and asked to determine which lottery a given reason would select. Their responses were compared against the preferences produced by the formal rules. (*D*) LLMs accurately identify decision reasons in verbal reports: the best-performing model from (*C*), Qwen-3-235B-A22B, was used to evaluate each reason’s presence in participants’ verbal reports and assign confidence judgments (0 to 100). Validation involved checking alignment between reason-implied choices and participants’ actual choices. A confidence threshold of T=80 was determined to optimize accuracy (94.7%) while minimizing the proportion of trials without identified reasons. (*E*) Variability of identified reasons across participants and problems: the reasons identified in (*D*) were aggregated into reason-by-participant and reason-by-problem matrices, serving as the basis for subsequent analyses of heterogeneity in reasoning patterns.

Using choices between pairs of monetary lotteries (Fig. 1 *A* and *B*), we show that LLMs can accurately evaluate monetary lotteries and various candidate decision reasons (Fig. 1*C*); that LLMs can accurately identify decision reasons in empirically gathered verbal reports, leading to accurate predictions of choices (Fig. 1*D*); and that our approach can reveal key insights into the decision reasons people use, both generally and conditional on the situation (Fig. 1*E*). Finally, we introduce a predictive model based on the identified distributions of decision reasons that reaches a level of predictive accuracy on par with cumulative prospect theory, a common benchmark, in predicting human risky choice.

Our investigation establishes an LLM-driven analytic framework for analyzing human decision making based on decision reasons and verbal reports. On its basis, we find that verbal reports have epistemic value: they contain information that makes it possible to explain and predict choices beyond what can be achieved by analyzing choices alone. We also demonstrate that, in contrast to the assumption of invariance, there is substantial variation in decision processes. This variation arises primarily from the structure of choice problems and conflicts with the common classification of choice problems based on outcome domain and certainty, revealing insights into the link between decision situations and processes.

## Results

The results are organized into four main parts. First, we briefly introduce the risky choice paradigm and define a formal set of candidate decision reasons. We then evaluate how accurately various LLMs can determine, for a given reason, which option is preferred across benchmark choice problems. Second, we apply the best-performing model and our decision framework to participants’ free-text verbal reports to classify the reasons underlying their decisions. To validate these classifications, we examine the alignment between the choices implied by the reasons and the actual choices of the participants. Third, we analyze the marginal distribution of the identified reasons to determine which were the most relied on. Fourth, we explore how the distribution varies between individuals and choice problems, uncovering systematic heterogeneity and clustering that diverge from conventional typologies. Finally, we demonstrate that the distributions of decision reasons yield equal or higher out-of-sample prediction accuracy of choices than prospect theory.

### LLMs Accurately Evaluate Decision Reasons and Choice Problems.

To effectively use LLMs to identify prespecified decision reasons in verbal reports, it is crucial to establish that they can accurately process both choice problems and the reasons behind them. We therefore begin our analysis by testing how well LLMs can predict the choices that result from applying a specific decision reason to a choice problem. As mentioned, choice problems here involve choosing between two monetary lotteries, each offering different monetary outcomes with different probabilities. An example is the choice between the following two lotteries:

$$\begin{array}{cc} & \text{Lottery A}:\left\{\text{€}6,000\;\text{with}\;0.1\%\;\text{and}\;0\;\text{with}\;99.9\%\right\}\\ & \text{Lottery B}:\left\{\text{€}3,000\;\text{with}\;0.2\%\;\text{and}\;0\;\text{with}\;99.8\%\right\} \end{array}$$

We further define decision reason as a rule with two components: 1) the reason specifies which outcome and probability are considered and how to transform them, and 2) a decision rule that discriminates between the lotteries based on the information considered. For example, the two components for the *most probable outcome* reason were 1) *The reason considers the most probable outcomes.*; and 2) *The reason prefers the lottery with the more favorable most probable outcome.*

Following this definition, we developed a set of 47 reasons using two approaches (*Decision Reasons* and *SI Appendix*, Table S1). First, we include several reasons based on basic elementary attributes of the choice problems, such as minimum outcome, maximum outcome, or a higher outcome range. Second, based on a list of computational models of risky choice from a recent review (39), we specified one or more decision reasons to capture the core concepts embodied by each model. For example, we specified several decision reasons to capture concepts from prospect theory, such as loss aversion, overweighting of small probabilities, and sure outcome presence.

For each decision reason, we produced two representations (see Fig. 1*A* for examples): a verbal decision reason that captures the relevant pieces of information and how this information is used to make a choice (Fig. 1*A*, *Left*) and a formal decision reason that specifies a formal decision rule that can be applied to a choice problem to identify the reason’s preferred lottery (Fig. 1*A*, *Right*). Having both a verbal and a formal representation was necessary to evaluate whether an LLM can correctly interpret decision reasons expressed in natural language in the context of monetary lotteries.

**Fig. 2.**
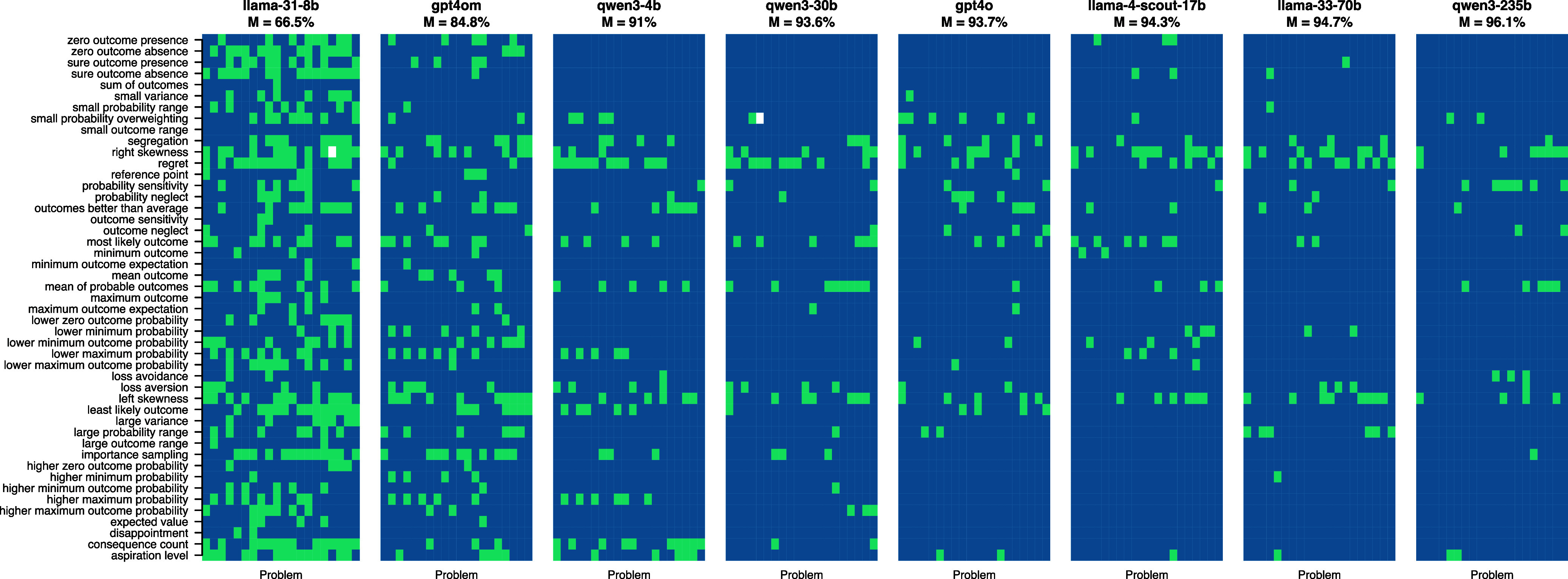
LLMs’ ability to understand choice problems and decision reasons. Each tile indicates whether the LLM identifies the lottery preferred by a decision reason (y-axis) in a choice problem (x-axis). Blue indicates correct LLM responses, green indicates incorrect ones, and white shows instances in which model output was erroneous (e.g., LLM returned a repeated sentence). Panel titles display the names and accuracies of LLMs.

To evaluate whether LLMs can accurately interpret and apply verbal decision reasons, we used a collection of 20 choice problems (*Choice Problems* and *SI Appendix*, Table S2). Of these, 16 canonical problems were used to develop prospect theory. We added four additional problems to capture decision making for options with both positive and negative outcomes. For each choice problem, we presented different LLMs with information about the problem and a decision reason and asked them to determine which option the decision reason prefers (*Prompt Engineering*). We considered a total of 8 LLMs, including GPT-4o (40) and leading open-source models from the Qwen (41) and Llama (42) families. Fig. 2 presents the results of this analysis. The best-performing model was Qwen3-235B-A22B, which accurately identified the preferred option in over 96% of cases.

In sum, our analysis indicates that current LLMs can accurately evaluate verbally expressed decision reasons in the context of monetary lotteries, with only rare exceptions.

### LLMs Accurately Identify Decision Reasons in Verbal Reports.

Having established that LLMs accurately evaluate monetary lotteries and the set of decision reasons we developed, we now investigate whether LLMs can accurately identify these decision reasons in verbal reports from human participants. We conducted two empirical studies with slightly different instructions for participants. As this did not affect the results (*Behavioral Experiment*), we present the findings of the two studies together (N=86). Participants made incentivized decisions for each of the 20 choice problems and afterward reported how and why they made their choice using a free-text response box (Fig. 1*B*). Participants received minimal instructions (*Behavioral Experiment*) and produced short texts with a median = 27 words (IQR = 26). The choices made by the participants aligned closely with the results of a recent replication of the preference patterns implied by prospect theory (43) (*Behavioral Experiment*), indicating that the free-text reporting procedure did not systematically bias participants’ preferences.

To identify the predefined decision reasons in the verbal reports, we designed a multistage prompt (*Prompt Engineering*) that asked the LLM to perform a series of evaluations. First, it assessed whether the reason could be applied to the choice problem by checking if the relevant information was present. Second, it evaluated and summarized participants’ justifications for their choice. Third, it assessed whether the individual used a given decision reason by comparing the information relevant to the reason with the individual’s justification. Finally, it provided a continuous judgment of confidence between 0 (certain the decision reason was not used) and 100 (certain the decision reason was used), with 50 indicating indifference. We performed this analysis using four selected open LLMs of different sizes. By using open models, we circumvented potential privacy issues that could arise from sharing free-text responses with a proprietary overseas service (44). In the main text, we report the results from the best-performing model, Qwen3-235B-A22B. See *SI Appendix*, Fig. S3 for comparisons between the four models.

As a first step in validating the LLM’s ability to identify the reasons, we investigated whether its numerical judgments of a reason’s presence or absence were consistent with people’s choices. Here, we again drew on the formal decision rule for each reason. If a participant applied an identified decision reason, their choice should align with the choice implied by that reason’s formal rule. To evaluate this, we tabulated the LLM’s confidence judgments as a function of whether the actual choice and the choice implied by a given decision reason were aligned.

**Fig. 3.**
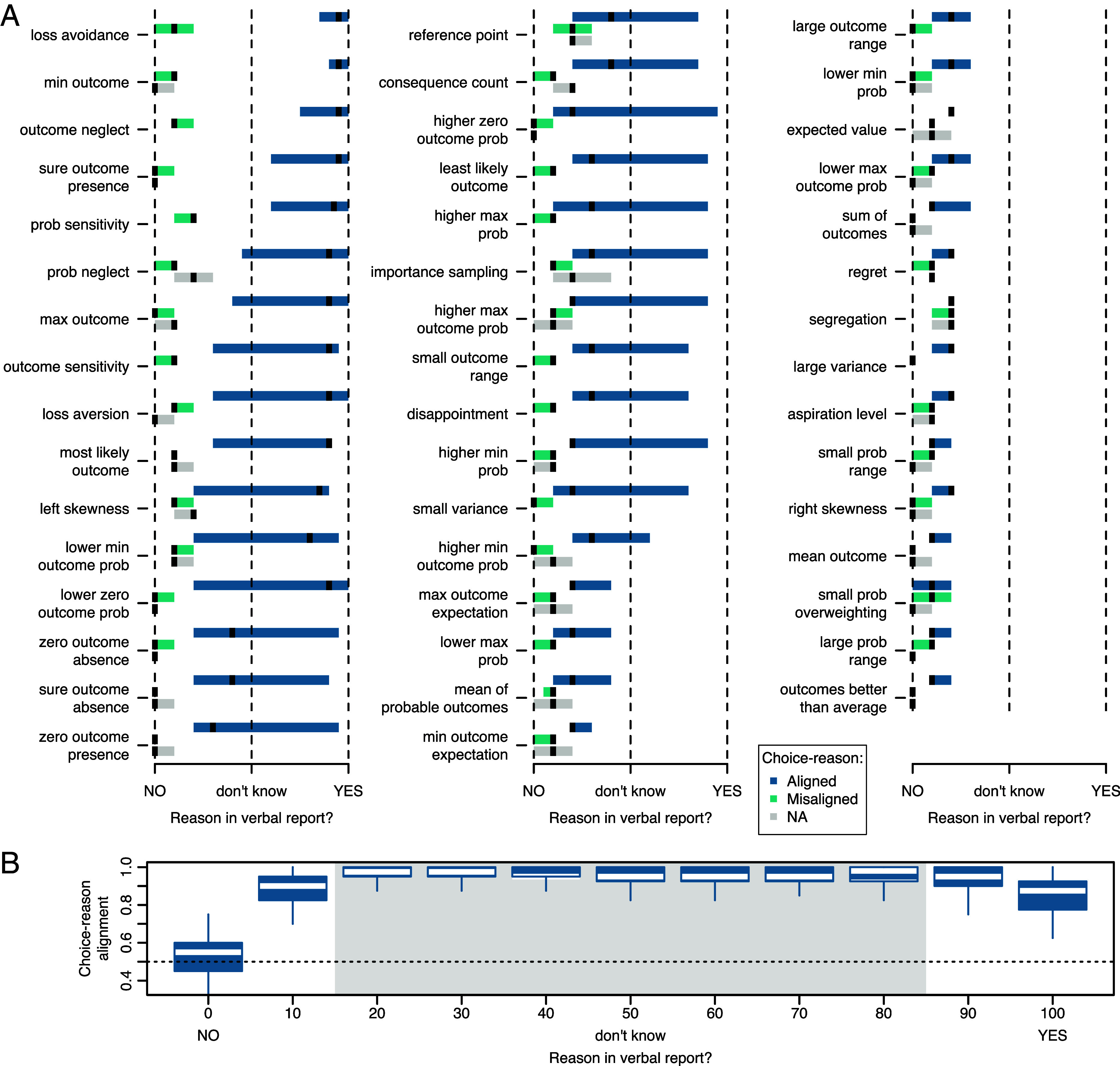
Evaluation of the LLM’s (Qwen3-235B-A22B) identification of decision reasons. (*A*) Distributions of LLM confidence assessments (0 to 100) for decision reasons as a function of alignment between reason-implied preferences and actual participant choices. Blue indicates aligned cases, i.e., a reason-implied preference matched participant choice; green indicates misaligned cases, i.e., a reason-implied preference did not match participant choice; gray indicates indifference cases, i.e., a reason had no preference between lotteries because it could not be applied or did not discriminate for a given problem. Box edges show first and third quartiles; the bold line indicates the median. (*B*) Alignment of decision reason preferences and choice as a function of the confidence threshold used for determining reason presence. For each threshold level, decision reasons were identified for each trial based on LLM assessments equal to or greater than the threshold. Identified reason preferences were then determined using majority rule for each trial and compared against observed choice (with random preferences in case of ties or no identified reasons). The boxplots summarize distributions of individual alignments across participants. The threshold T=80 was selected as optimal because it maximized the LLM’s confidence and alignment (94.7%), while minimizing the proportion of trials with no identified reason (3.4%).

Fig. 3*A* shows the distribution of judgments for each decision reason, separated into aligned (blue) and misaligned (green) cases, as well as cases where the reason could not discriminate between lotteries (gray), for example, because the key pieces of information used by the reason are not available in the lottery (e.g., the maximum outcome reason requires a difference between the maximum outcomes of both lotteries). The numerical judgments of the LLM are, on average, higher when the actual and implied choices aligned (Fig. 3*A*, *Correct*) than when they were misaligned (Fig. 3*A*, *Incorrect*). In general, for aligned choices, 37% of the judgments exceeded the indifference level of 50, while only 2.4% did so for misaligned choices. The low percentage for misaligned choices is particularly important, as in these instances, it should be impossible for the reason to have been present. This contrasts with aligned choices, where actual and implied choices could have aligned coincidentally, as different reasons can imply the same choice. These results demonstrate that the LLM’s judgments successfully discriminate between present and absent reasons.

Next, we estimated how well the LLM identifies the reasons reflected in verbal reports. This required setting a threshold for confidence judgments to determine if a reason was identified. Since we do not know the model’s calibration, we evaluated a sequence of thresholds across the full confidence range from 0 to 100. For each threshold value and separately for each choice trial, we recorded which decision reasons were assigned values equal to or higher than the threshold. Then, for each individual choice, we generated a predicted preference based on the majority preference within the identified decision-reason set (*Choice Prediction*). If no reasons were identified above the threshold or the majority rule was inconclusive, the model made a random choice.

Fig. 3*B* shows the alignment of the decision-reason majority-rule model at each threshold level. As can be seen, threshold levels of T∈20,...,80 yielded the highest average accuracy, ranging from 95 to 96%. The substantial increase in accuracy from the threshold T=10 onward indicates that the LLM was especially accurate in identifying cases where a reason was definitely not used. The decrease in accuracy at high thresholds of T=90 and T=100 is due to a substantial proportion of trials with no identified reason (4.9% and 25.1%, respectively). Based on these results and the principle of parsimony, we selected a threshold of T=80 to identify the presence of decision reasons in subsequent analyses. This threshold allowed for an identification of at least one reason for 96.6% of trials, identified a median of seven reasons per trial ($Q_{1}=4$; $Q_{3}=9$), and achieved an alignment between preferences of identified reason and choice of 95%.

In sum, our results demonstrate that LLMs can accurately identify reasons from verbal reports that match participants’ choices, and thus likely reflect the processes underlying those choices.

### LLMs Reveal the Relative Importance of Decision Reasons.

Using the decision threshold identified in the previous section, we can analyze which decision reasons are most characteristic of people’s choices overall.

**Fig. 4.**
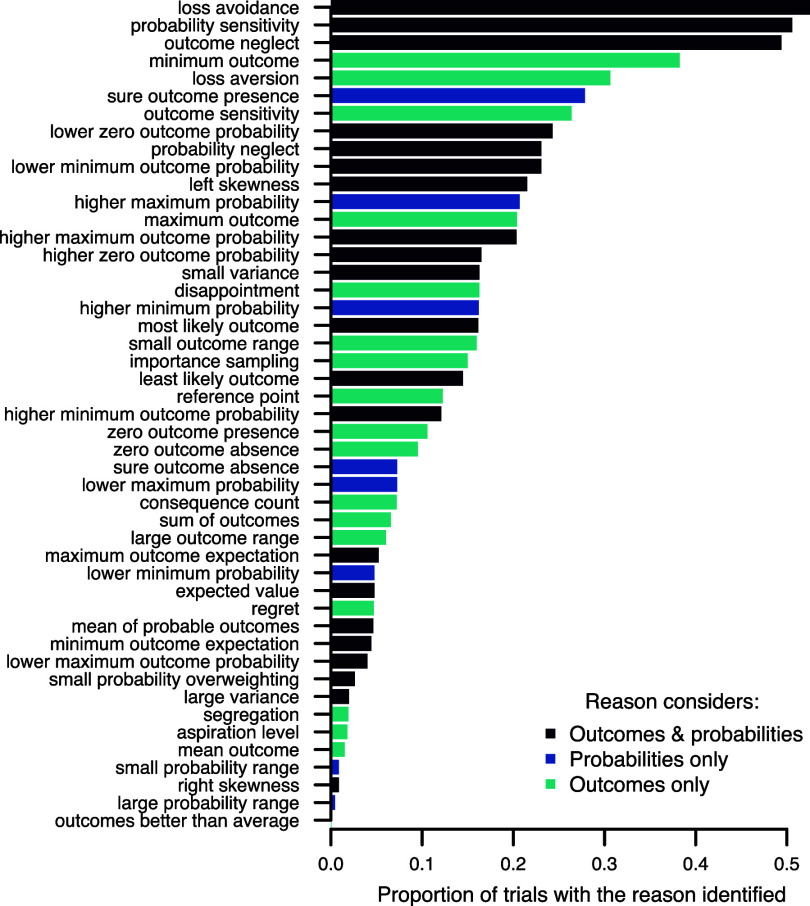
Identified decision reasons. The y-axis is the proportion of choice trials for which a given decision reason was identified (i.e., LLM confidence that the reason was used was equal to or greater than 80).

Fig. 4 shows the prevalence of reasons, defined as the proportion of trials for which each reason was identified. The reasons are colored according to whether they consider the outcomes (green), probabilities (blue), or both (black). Several notable results emerge. First, the specific problem information a reason considers has no impact on its prevalence. Second, the most prevalent reasons imply risk aversion or a desire to come out ahead. These reasons prefer options with a lower probability of a loss (loss avoidance), a higher probability of favorable outcomes (outcome neglect), a more favorable minimum outcome (minimum outcome and loss aversion), or a sure outcome (sure outcome presence). Third, many more complex decision reasons, including those reflecting major concepts such as reference points, regret, or expected value, are considerably less prevalent. This may suggest that these concepts are less related to people’s decisions than previously thought. However, another possibility is that these reasons are difficult to express in verbal reports and equally challenging for the model to match. In fact, we observed a weak negative correlation between the length of the verbal reports and the frequency with which the reasons were identified (r=−0.23)

In sum, our results show that people’s decision making relies on reasons that consider both outcomes and probabilities and favor risk-averse decision making focused on coming out ahead.

### LLMs Reveal Individual and Contextual Variability in Decision Reasons.

**Fig. 5.**
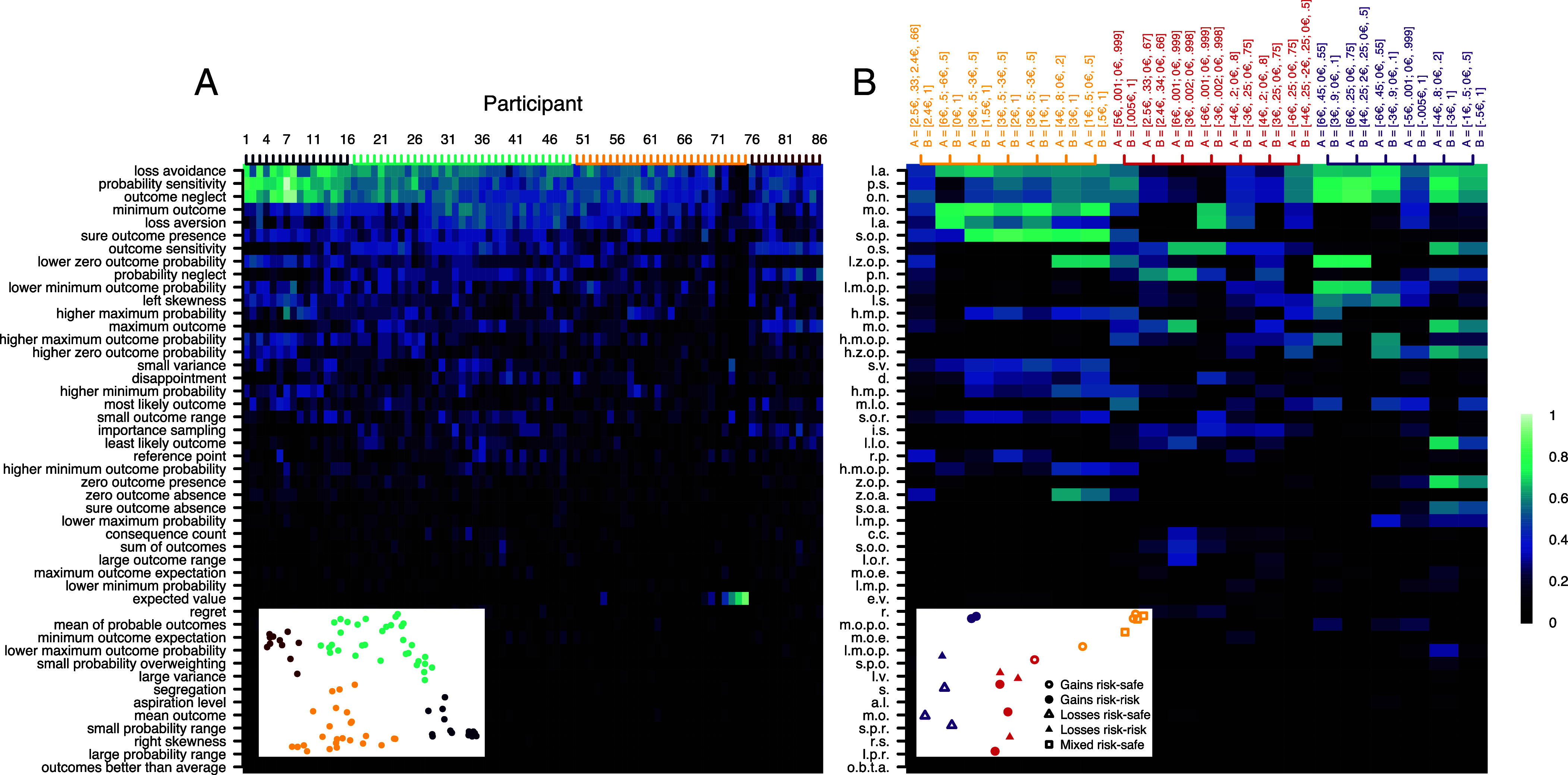
Variability of identified decision reasons across individuals and problems. (*A*) Proportions of choice trials in which a given decision reason (y-axis) was identified for a given participant (x-axis). The *Inset* shows PaCMAP projection of participants’ reasons distributions into a two-dimensional space, based on data from panel (*A*). (*B*) Proportion of choice trials in which a given decision reason (y-axis) was identified for a given choice problem (x-axis). The *Inset* shows PaCMAP projection of problems’ reasons distributions into a two-dimensional space, based on data from panel (*B*). The colors assigned to participants/problems in (*A*) and (*B*) represent distinct clusters identified via hierarchical clustering with the Ward method based on the PaCMAP projections (*Clustering*).

Next, we directly tested the invariance assumptions that characterize and constrain existing computational accounts of risky choice. We do this by examining whether the profiles of decision reasons vary systematically across participants and choice problems. We calculated, for each participant (Fig. 5*A*) and each choice problem (Fig. 5*B*), the proportion of trials in which each decision reason was identified, yielding two reason-by-case proportion matrices visualized as tile plots.

To visualize the structure of the participant- and problem-specific reason profiles, we applied a nonlinear dimensionality reduction algorithm (45, PaCMAP) to each matrix, producing two-dimensional embeddings that reflect the distances between the respective profiles (Insets of Fig. 5 *A* and *B*). We then clustered the embeddings using hierarchical clustering and used these clusters to organize the columns of the tile plots and to color the two-dimensional scatter plots. This revealed a visual grouping of similar participant- and problem-specific reason profiles (*Clustering*). We chose to extract only four clusters for participants and three clusters for problems to avoid overfitting and to provide a parsimonious description of the differences in participant and problem profiles.

Participant profile clusters showed considerable overlap. All four clusters exhibited reliance on the same core set of reasons—loss avoidance, probability sensitivity, outcome neglect, and minimum outcome—and differed primarily in the overall intensity of reason activation rather than in qualitatively distinct reasoning strategies. The first cluster (purple, Fig. 5*A*) comprised a small group of participants with a somewhat more pronounced reliance on probability-related reasons, such as probability sensitivity, outcome neglect, and higher maximum probability, suggesting a relatively greater sensitivity to the likelihood of outcomes. The second cluster (cyan), encompassing the majority of participants, showed a similar profile, but placed comparatively more weight on outcome-related reasons, including minimum outcome, loss aversion, outcome sensitivity, and probability neglect, suggesting a somewhat stronger focus on the magnitudes of potential gains and losses. The third cluster (yellow) was again broadly similar, but presented a more diffuse profile, blending both probability- and outcome-related reasons without a clear distinguishing emphasis, except for a few participants relying specifically on expected value. The fourth cluster (brown) exhibited the most diffuse pattern. Overall, however, these differences between clusters were subtle and should be interpreted cautiously.

The analysis of problem profiles revealed clusters with clearer characteristics. A first cluster of problems (yellow) exclusively includes problems with a sure option. These problems are primarily associated with reasons that favor the sure option, such as sure outcome presence, but also reasons that promote risk aversion, including probability sensitivity, minimum outcome, or small variance. Notably, this also includes loss avoidance, which prefers options with a lower probability of a loss, even though some of these problems do not involve losses. This is likely because in these cases, a zero outcome is interpreted as a loss by both humans and the language model. A second cluster of problems (red) features almost exclusively risky–risky problems with similar probability distributions and is thus primarily associated with outcome-related reasons. Prominent reasons include outcome sensitivity, probability neglect, and maximum outcome. A third cluster of problems (violet) encompasses problems that could be considered intermediate between the other two clusters. They mostly include risky–risky and risky–safe problems with probability distributions that are more distinct and, in some cases, approach a trade-off between risk and relative certainty. These problems are less consistently associated with decision reasons than in the other two clusters. However, the problems appear to share mostly the risk-averse reasons present in the first cluster, such as probability sensitivity, loss avoidance, and outcome neglect. Overall, these results reveal a clear clustering of problems by reason profiles that appears to be primarily shaped by the problems’ probabilistic structure. In particular, this clustering diverges from the canonical classifications in decision science, which focus on the domain (gain, loss, or mixed) and the type of problem (risky–risky vs. risky–safe).

We performed two analyses to quantify the amount and source of variability. To provide an easily interpretable estimate, we calculated the average pairwise shared variance (mean $r^{2}$) among participant and problem profiles, yielding values of 0.57 for participants and 0.23 for problems. The high shared variance among participants confirms the impression from the cluster analysis: participant reason profiles were largely similar to one another, with differences reflecting gradual shifts in emphasis rather than qualitatively distinct strategies. Problem profiles, by contrast, were far more heterogeneous. A Bayesian hierarchical regression (*SI Appendix*, Table S3) corroborated this pattern, with the variance of the reason-by-problem interaction (3.58) exceeding that of the reason-by-participant interaction (0.32) by more than an order of magnitude, indicating that the choice of decision reasons was primarily shaped by the structure of the problem rather than by individual differences.

Together, these results point to substantial reason variability, in particular among problems, with problem groups emerging that do not align with canonical problem classifications in decision science.

### Evaluating the Predictive Accuracy of Reason-Based Models.

In our final analysis, we conduct a critical test of our verbal report-based approach and evaluate which source of variability, participant or problem, helps more to predict people’s choices.

One common objection to verbal report-based approaches is that they do not reveal more about the decision process than can be extracted from the choice itself. Our framework allows for a direct test using verbal and formal reasons as the basis for separate predictive models in an out-of-sample prediction analysis. To achieve this, the dataset was randomly split into training and test sets in each of 5,000 iterations (an 80%–20% split, stratified by participant). From the training sets, we identified distinct sets of reason profiles and used them to predict the choices in the test set. Specifically, we treated the reason profiles as an ensemble model, where each reason’s choice prediction is weighted by its relative frequency in the profile and then aggregated. Crucially, we performed this separately for verbal and formal reasons. The verbal reason profiles are based on the reasons that the LLM identifies in the verbal reports, whereas the formal reason profiles are based on the reasons for which the formal reason’s prediction is aligned with choice. If verbal reports inform decision processes better than the choices themselves, verbal report-based reason profiles should outperform those based on formal reasons. In addition, they should achieve this performance using a more informed distribution of reasons. We test these assertions by calculating the out-of-sample accuracy and the average reason-distribution perplexity (*Choice Prediction*), which indicates the effective number of reasons relied upon to make a choice.

To simultaneously learn more about which source variation is most crucial, we considered several ways to determine the reason profiles. We implemented two baseline profiles: one based on a uniform distribution of reasons and another on the marginal distribution of reasons in the training data. We then implemented six conditional reason profiles to pinpoint important sources of variation. These profiles consider the conditional distribution of reasons in the training data based on individual participants, clusters of participants, individual problems, clusters of problems, and canonical problem classes (crossing problem type and domain). We posited that if the participant and problem variation are relevant, then using their respective profiles should yield a higher predictive accuracy than either baseline. As a benchmark to evaluate the observed predictive performance, we also implemented two versions of prospect theory.

**Fig. 6.**
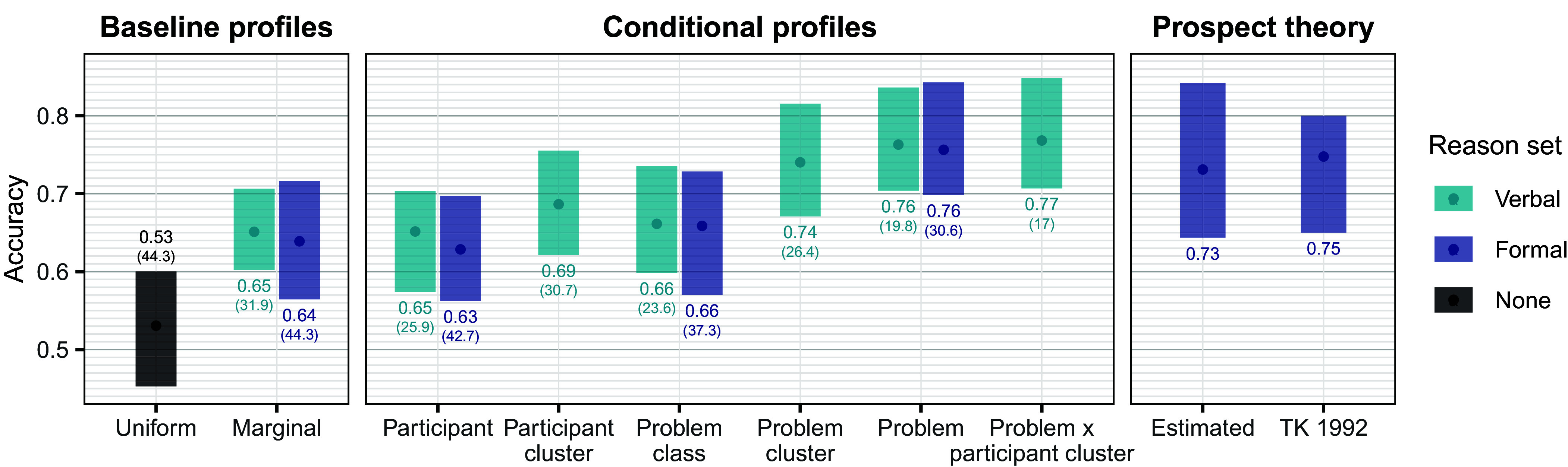
Out-of-sample prediction accuracy based on conditional distributions of LLM-identified decision reasons. In each of 5,000 iterations, the dataset was randomly split into training and test sets (an 80%–20% split stratified by participants). From the training data, we computed the frequency with which each decision reason was identified within a given condition (e.g., for each problem, cluster, or participant), yielding condition-specific distributions of reasons. These were combined with a fixed reason-preference profile for each choice problem to generate predicted choices in the test set by computing the weighted average of reason-implied preferences. For each run, prediction accuracy was calculated separately for each participant in the test set. The boxes show first and third quartiles, and the points are means of the individual mean accuracies, aggregated across all 5,000 iterations. Prediction models differ by the type of reason used, with blue indicating models based on verbal reasons and purple models based on formal reasons. They also differ by how the reason distributions were conditioned: Uniform—deterministic predictions based on the predefined reason-preferences of all reasons (no conditioning); Marginal—conditional on the overall frequency of reason use across the dataset (cf. Fig. 4); Participant & Participant cluster—conditional distributions derived per participant or participant cluster (cf. Fig. 5*A*); Problem class—canonical classification into five groups: risky–safe gains, risky–risky gains, risky–safe losses, risky–risky losses, and risky–safe mixed; Problem & problem cluster—conditional distributions derived per problem or problem cluster (cf. Fig. 5*B*); Problem × participant cluster—conditional distributions derived for combinations of individual problem and participant clusters; Estimated/TK 1992—benchmark out-of-sample performance of prospect theory, either estimated from our data or using parameter values from the literature. The numbers show the mean OOS accuracy and mean perplexity scores of the condition-specific reason distributions. See *Choice Prediction* for details.

Fig. 6 shows a comparison of the out-of-sample performance. We see that all reason profiles outperformed the uniform profile, establishing that the relative distribution of reasons contains information about people’s choices. Regarding the type of reason, verbal reason models (blue) have a slight but consistent advantage of 0 (problem) to 3.5 (participant) percentage points over those based on formal reasons (purple). Note that comparisons are only available for conditions that do not involve clusters determined from verbal reasons. Bayesian hierarchical beta regressions yield posterior probabilities ranging from 0.81 (marginal) to 0.97 (participant) for differences favoring verbal reasons (see *SI Appendix*, Table S4). Crucially, verbal reports achieved this advantage with substantially lower perplexity. On average, the perplexity of verbal reasons was 24.7, as compared to 40.3 for formal reasons. This means that the verbal-reason models achieve equal or higher performance while relying on 15.6 fewer reasons, with all posterior estimates of the differences credibly below zero (*SI Appendix*, Table S5). The verbal reasons thus provide a parsimonious and diagnostically informative representation of decision processes, filtering out reasons that may coincidentally align with choice but are not part of the decision process.

Concerning the different profiles, participant cluster (${\text{M}}_{\mathrm{acc}}$ = 68.6%), problem cluster (74%), problem (76.3%), and problem by participant cluster (76.8%) outperformed the marginal distribution (65.1%), but not participant (65.1%) and problem class (66.1%). Consistent with the low variability in reasons among participants and the variability in reasons for problems that are inconsistent with canonical problem classes (Fig. 5), the participant and problem-class conditions provide little information for predicting people’s choices. Furthermore, the problem condition, as well as the problem by participant-cluster condition, showed the highest predictive performance, exceeding the performance of two versions of prospect theory: one estimated from our data (73.1%) and one using the parameters from Tversky and Kahneman’s seminal paper (74.8%; 8). As prospect theory was designed to capture the modal choice patterns of the problems in our study, and these patterns also empirically align with those of a recent large-scale replication of the original work (43), these performances are notable. They suggest the presence of unaccounted-for problem variation in traditional models of risky choice. In addition, they suggest that problem variability, by itself or in conjunction with participant clusters, is most helpful for predicting choice.

In sum, our results reveal that verbal reasons paired with LLM identification from verbal reports predict people’s choices better than formal reasons aligned with choice and do so while providing more insight into the underlying decision process. This advantage of verbal reports seems to be mainly due to greater variability in reasons across problems, suggesting that choice is driven by the probabilistic structure of choice problems.

## Discussion

Our analytical framework and empirical results demonstrate that large language models can be leveraged to automatically analyze verbal reports and help uncover the elements of human choice. We first established that LLMs can accurately interpret the formal structure of monetary lotteries and a diverse set of 47 decision reasons. Applying this capacity to free-text reports from human participants, we found that LLM-identified reasons aligned with actual choices in 95% of trials, validating the method’s accuracy. The analysis revealed that the most prevalent decision reasons are those that favor risk aversion and the desire to come out ahead, such as focusing on the probability of winning or avoiding a loss. Critically, we found substantial variability in the use of these reasons. This, in turn, was driven largely by problem variability. A predictive model built on these problem-specific reason profiles outperformed a popular benchmark model of risky choice, prospect theory, used to account specifically for the kinds of choice problems studied here. Together, these results establish that LLM-based analysis of verbal data is a viable and scalable method for decision research.

Our findings both align with and challenge the dominant theories of choice. The prevalence of reasons geared toward risk aversion and achieving favorable outcomes resonates with the core tenets of models like prospect theory (8), as well as with other accounts such as satisficing or aspiration level theories (39, 46). However, our central finding, that decision reasons are not stable but vary systematically with the problems’ structure, implies that the invariance assumptions common to many formal models are misplaced. This result is strongly supported by recent large-scale studies, which find that context-dependent models provide the best predictive power (13, 23). Such models succeed precisely because they learn to apply different strategies in different contexts. Our work empirically grounds this context dependence on people’s articulated reasons in a highly interpretable manner, paving the way for more explicit models of how the environment, here in terms of the structure of the problems, triggers shifts in decision processes.

Methodologically, our results demonstrate that verbal reports are a rich and valid source of data, supporting Simon and Ericsson’s view on the validity of verbal reports in inferring cognitive processes (38, 47). The high alignment between reasons extracted from verbal reports and actual choices provides evidence of the predictive power that self-reports can offer (48, 49). This work joins a growing body of evidence that indicates that subjective stated preferences are often more reliable and stable than “revealed” preferences inferred from choice behavior alone (10, 50, 51). A complementary analysis (*SI Appendix*) further corroborates the epistemic value of verbal reports: predicting choices directly from trial-level representations of the reports achieves 84% out-of-sample accuracy from raw embeddings of the report text and 93 to 95% from the LLM-identified decision reasons. This reinforces that talk is far from cheap: verbal reports convey nearly complete information about the accompanying choice. We emphasize, however, that our main approach deliberately goes beyond such prediction: by aggregating identified reasons into reusable participant- and problem-specific profiles, our approach yields interpretable decision-process components that generalize to new trials for which no verbal report is available.

Our work also contributes to recent studies that show the value of LLMs in uncovering the decision process (52). Past work has found that large language models provide powerful predictive models (4), propose new theoretical models of choice (53), help analyze social decision instructions (54), and map the landscape of the literature on behavioral decision making (55). In general, this work highlights the potential LLMs have to advance the cognitive and decision sciences.

Beyond validating a method, the decision-reason approach can serve as a diagnostic device for scientific discovery. By systematically cataloging the reasons used in various contexts, our approach can help identify weaknesses in existing theories and guide the development of new, more nuanced ones. The approach is highly adaptable, permitting the specification of an arbitrary number of alternative reasons tailored to the domain of study. The use of open-ended verbal reports, which allow participants to voice their reasoning in rich and flexible ways (38, 48), could be applied to uncover the processes of choice in other domains, such as intertemporal, moral, or social decision making.

Limitations of the present study should be acknowledged. First, our analysis was based on a set of 20 choice problems. Although canonical, the problem set likely underestimates the full range of reasons people might use across a wider range of contexts. Second, our sample of 86 participants is modest by current standards; however, many key analyses occurred at the trial level (N = 1,720) or were independent of the empirical data, and the out-of-sample prediction comparison was supported by statistical tests across 5,000 iterations. Third, while our method achieves competitive or superior out-of-sample prediction accuracy relative to a prominent benchmark theory, we do not formalize a new, integrated theory of strategy selection. Fourth, the study was conducted with German-speaking participants, and the universality of these reasoning patterns remains to be tested. Finally, the act of generating a verbal report might itself influence cognition. Retrospectively explaining a choice is a reflective, reconstructive process that can differ from the thoughts that occur concurrently during the decision (38).

In conclusion, this study successfully developed and validates a scalable method to identify the reasons behind human choices by applying LLMs to verbal reports. Our findings challenge the field’s foundational invariance assumptions. We demonstrate that decision strategies are not fixed but are deployed flexibly in response to the structure of choice problems. By bridging the gap between stated reasons and observed choices, this approach moves beyond relying solely on behavioral data and opens an avenue for developing more psychologically realistic context-sensitive models of human decision making.

## Materials and Methods

### Decision Reasons.

To characterize the decision making of participants from their verbal reports, we defined a structured set of 47 candidate decision reasons. Each reason is an operational construct designed to capture a potential strategy or principle for choosing between risky options. In our framework, a decision reason must meet a minimal discriminability criterion: it must specify a consistent preference for one lottery over another based on identifiable features of the choice problem. This requirement led us to exclude vague heuristics, such as the “affect heuristic,” that lack a quantifiable basis for choice.

Our final set of reasons was derived from two sources. First, we defined 27 reasons based on elementary properties of the lotteries. These included singular (e.g., maximum outcome, minimum probability) and composite features (e.g., outcome range, variance). We verbalized each of these using a two-part format: the first part specifies the information considered (e.g., The reason considers the maximum outcome of each lottery), and the second describes the resulting preference (e.g., The reason prefers the lottery with the more favorable maximum outcome). For features where the direction of preference could be context-sensitive (e.g., maximum probability), we defined directional variants as separate reasons (e.g., higher maximum probability vs. lower maximum probability) to ensure verbal representations remained unambiguous.

Second, we derived 20 additional reasons by analyzing formal decision models and preference patterns reviewed in ref. 39. We distilled unique, verbalizable principles from each model, abstracting away technical details. For example, while participants are unlikely to articulate concepts like nonlinear outcome transformations, we captured the underlying logic with reasons such as outcome sensitivity (where outcome differences matter) and outcome neglect (where they do not). We applied a similar logic to formulate reasons that reflect nonlinear probability weighting, loss aversion, and regret. This set of 47 reasons is not intended to be exhaustive but serves as a systematic and scalable foundation for identifying decision reasoning in verbal data. A complete list is available in *SI Appendix*, Table S1.

### Choice Problems.

We used 20 monetary choice problems, 16 of which were adopted from the foundational paper on Prospect Theory by Kahneman and Tversky (7). These problems are canonical in the field and cover risky–safe and risky–risky formats across both gain and loss domains. To increase structural diversity, we supplemented this set with four mixed-domain problems. Each problem involved a choice between two lotteries with up to three outcomes. A full specification is provided in *SI Appendix*, Table S2 (see also Fig. 5*B*).

### Prompt Engineering.

We used a zero-shot prompting approach for all LLM tasks. In this approach, the model receives instructions without examples, relying only on carefully designed prompts. Our prompts were designed in accordance with established best practices. Key features included: assigning the model an explicit role (e.g., “You are a decision analyst”), providing clear definitions of the task components (i.e., choice problem, decision reason, verbal report), and incorporating chain-of-thought (CoT) instructions to foster structured, step-by-step reasoning (56). CoT prompting encourages the model to generate intermediate steps before its final conclusion, which often improves accuracy and interpretability. For the main task of analyzing verbal reports, the prompt explicitly instructed the model to ignore any mention of the participant’s choice (e.g., “I chose A”) and to focus solely on the expressed reasoning. The complete prompts are provided in *SI Appendix*, Figs. S1 and S2. The LLM’s outputs consistently followed the specified format, which allowed for straightforward programmatic extraction of the final answers.

The prompt is the result of several iterations, informed by at least three major insights. First, performance benefited from running separate prompts for each reason, rather than running all reasons simultaneously. Running the reasons separately prevents responses to earlier reasons from influencing responses to later reasons; however, this also substantially increases computational costs. Second, providing the problem in the prompt and instructing the LLM to first check whether a reason even applies to a problem reduced model hallucinations, especially when combined with chain-of-thought reasoning. Third, providing a scale midpoint significantly helped with calibration, resulting in better use of the entire scale and more interpretable low and high judgments.

### Inference with LLMs.

For the initial model comparison, we used both proprietary (GPT-4o, GPT-4o-mini) and open-weight LLMs. The proprietary models were accessed via the Azure API at the Max Planck Institute for Human Development. Open-weight models (Llama, and Qwen) were run locally on a high-performance computing cluster using Hugging Face (see, ref. 1, for a tutorial) and vLLM (https://docs.vllm.ai/). All models were run with default inference settings (e.g., temperature) to assess their typical performance.

Critically, all verbal reports were analyzed in their original German without any preprocessing, cleaning, or translation. This included typographical errors, grammatical inconsistencies, and informal phrasing, allowing us to evaluate the LLM’s ability to handle raw, naturalistic text.

For the main analysis, we selected Qwen3-235B-A22B. This model achieved the highest accuracy in our initial validation (Fig. 2), outperforming all other tested models. Its open-weight and multilingual nature made it an ideal choice for analyzing the German-language reports in a transparent and reproducible manner. The complete codebase and model outputs are available in our project repository.

### Behavioral Experiment.

The study was conducted in person in the laboratories of the Max Planck Institute for Human Development in Berlin. A total of 86 adults (M = 28.4, SD = 6.2; 62% female; 50% university degree) participated after giving informed consent. The study was approved by the Institutional Review Board (Ethics Committee) at MPI for Human Development. Participants were compensated with a base payment of 17 euros plus a performance-based bonus (up to ±3 euros) determined by playing out one randomly selected choice trial for real money.

Participants completed 20 monetary choice problems in a randomized order. The task was presented on a computer using an interactive interface where lottery outcomes and probabilities were initially hidden and could be revealed by mouseover. After making their choice in each trial, a text box appeared with the following prompt:

*Bitte beschreiben Sie WIE und WARUM Sie diese Entscheidung getroffen haben. Wir möchten erfahren, mit welchen Schritten Sie Informationen gesammelt haben und welche Gründe Sie für Ihre finale Entscheidung in Betracht gezogen haben im Hinblick auf die Eigenschaften der Lotterien. Geben Sie die Schritte, wenn möglich, in der Reihenfolge an, in der sie stattgefunden haben.*

(English translation: Please describe HOW and WHY you made this decision. We would like to know what steps you took to gather information, and what reasons you considered for your final decision in relation to the characteristics of the lotteries. If possible, list the steps in the order they occurred.)

Due to a partial loss of early source code, the exact prompt phrasing for the first 41 participants is unavailable, but it was effectively equivalent. The correlation between the proportions of trials in which a given reason was identified across sessions was r=0.98, with a very small average absolute difference (M = 0.003, SD = 0.003), indicating that the distributions of identified reasons did not differ between sessions.

To validate our choice data, we compared the choice proportions for the 16 canonical problems to those from a large-scale replication study (43). The correlation between the choice proportions was very high (r=0.87), and the median absolute difference was small (Me=0.11), indicating that the addition of a verbal reporting task did not systematically distort decision behavior.

We collected a total of 1,720 free-text reports. The reports varied in length, with a median of 27 words ($Q_{1}=16$, $Q_{3}=42$) and 165 characters ($Q_{1}=96$, $Q_{3}=258$). The vast majority of participants provided substantive responses suitable for semantic analysis.

### Choice Prediction.

To evaluate whether the LLM-identified decision reasons could predict participants’ choices, we implemented two prediction strategies. First, we used a majority-rule approach to determine the optimal confidence threshold for identifying the presence of a reason (Fig. 3*B*). Second, we applied a reason-weighted aggregation model to test whether contextual distributions of reasons could generalize to unseen trials in an out-of-sample setting (Fig. 6). Together, these methods assess both the within-sample alignment and the broader predictive utility of our approach.

#### Determining a threshold for reason identification.

To determine an appropriate confidence threshold for identifying decision reasons, we tested how well the reasons selected at different levels aligned with participants’ actual choices. Because multiple reasons could be identified for a single trial, we used a majority-rule aggregation to derive one predicted choice per trial.

First, we mapped choice options to numeric values: Option B was coded as −1, Option A as +1, and indifference (used only for reason-based preferences) as 0. Let $R_{T,i}$ be the set of decision reasons identified for trial i at threshold T. This set can be represented as a vector of reason-implied preferences, where each reason $r\in R_{T,i}$ is uniquely mapped to a single value $v_{r}\in \left\{-1,0,+1\right\}$ according to its formal rule.

Second, the model’s predicted choice for trial i at threshold T was then calculated as

$${\hat{Y}}_{T,i}=\text{sign}\left(\sum_{r\in R_{T,i}}v_{r}\right),$$

where sign(·) returns +1 if the sum is positive (predicting Option A), −1 if negative (predicting Option B), and 0 in case of a tie. To compute prediction accuracy, we compared the predicted choice ${\hat{Y}}_{T,i}$ with the observed choice $Y_{i}^{\text{obs}}$:

$$Y_{i}^{\text{align}}=\left\{\begin{array}{cc} 1 & \text{if}\;{\hat{Y}}_{T,i}=Y_{i}^{\text{obs}}\\ 0 & \text{if}\;{\hat{Y}}_{T,i}\neq Y_{i}^{\text{obs}}. \end{array}\right.$$

A tie ${\hat{Y}}_{T,i}=0$ was scored 0.5 to reflect a random guess.

Finally, we aggregated accuracy at the participant level by averaging each participant’s alignment scores across their 20 trials. We evaluated thresholds T∈{0,10,20,⋯,100}, as the LLM’s confidence scores were provided in discrete steps of 10. The resulting distribution of participant-level accuracies is shown as a boxplot for each threshold in Fig. 3*B*.

#### Out-of-sample predictions.

This analysis tested whether the distribution of identified reasons within a specific context (e.g., for a particular problem or participant) could predict choices for new trials from that same context. This assesses if the observed variability in reason use reflects a consistent and generalizable structure.

First, we constructed a binary reason-identification matrix $X\in {\left\{0,1\right\}}^{n\times R}$, where n=1,720 is the number of trials and R=47 is the number of reasons included in the final analysis set. An entry $X_{i,r}=1$ indicates that reason r was identified for trial i (i.e., LLM confidence ≥80); otherwise, $X_{i,r}=0$. Context data for each trial included participant ID, participant cluster (Fig. 5*A*), problem ID, problem type (canonical types, and problem cluster (Fig. 5*B*).

Second, the data were split into training and test sets using an 80–20 stratified split within each participant, resulting in 16 training and 4 test trials per participant per iteration. Let $D_{\text{train}}$ and $D_{\text{test}}$ denote the resulting subsets. For a given context C (e.g., participants, problems, or clusters) with levels $\left\{C_{1},\cdots ,C_{J}\right\}$, we then computed the frequency of each reason within the training trials of level $C_{j}$, defined as $I_{C_{j}}=\left\{i\in D_{\text{train}}:C\left(i\right)=C_{j}\right\}$. The frequency vector was given by

$$f_{C_{j}}\left[r\right]=\sum_{i\in I_{C_{j}}}X_{i,r},$$

yielding $f_{C_{j}}\in N^{R}$, which represents the count of identified reasons for level $C_{j}$.

Third, we defined a reason-preference matrix $R_{\text{pref}}\in {\left\{-1,0,+1\right\}}^{D\times R}$, where D=20 was the number of unique choice problems. Each entry $R_{\text{pref}}\left[d,r\right]$ specifies the preference implied by reason r in problem d: +1 for Option A, −1 for Option B, and 0 if indifferent. For each test trial $i\in D_{\text{test}}$, we used the corresponding condition level $C_{j}\left(i\right)$ and problem d(i) to compute the predicted choice:

$${\hat{Y}}_{i}=\text{sign}\left(\sum_{r=1}^{R}f_{C_{j}\left(i\right)}\left[r\right]\cdot R_{\text{pref}}\left[d\left(i\right),r\right]\right),$$

with ${\hat{Y}}_{i}=+1$ for Option A, −1 for Option B, and 0 for ties.

Finally, prediction accuracy was evaluated analogously to the threshold-based analysis: each test trial contributed 1 for a correct prediction, 0 for an incorrect prediction, and 0.5 for a tie. The participant-level mean accuracy was computed with these scores. This entire procedure was repeated for 5,000 random splits. The resulting distributions of mean participant-level out-of-sample accuracies are shown as boxplots in Fig. 6.

#### Perplexity.

To characterize how many reasons effectively contribute to each predictive model’s predictions, we calculated the perplexity of the context-specific reason distributions. Perplexity is an information-theoretic measure that captures the effective number of categories in a distribution: it equals 1 when all weight is concentrated on a single reason and equals the total number of reasons when weight is spread uniformly. Lower perplexity thus indicates a more concentrated, parsimonious reason distribution.

Specifically, within each out-of-sample iteration, the frequency vector $f_{C_{j}}$ for a given level $C_{j}$ was normalized to form a probability distribution $p_{C_{j}}$, defined as

$$p_{C_{j}}\left[r\right]=\frac{f_{C_{j}}\left[r\right]}{\sum_{r^{\prime }=1}^{R}f_{C_{j}}\left[r^{\prime }\right]}.$$

The perplexity $PP_{C_{j}}$ for each profile was then computed as

$$PP_{C_{j}}=2^{H(p_{C_{j}})},\;\text{where}\;H\left(p_{C_{j}}\right)=-\sum_{r=1}^{R}p_{C_{j}}\left[r\right]\log_{2}p_{C_{j}}\left[r\right]$$

is the Shannon entropy of the distribution. For each context C (e.g., problem cluster), we calculated the average perplexity across all its constituent levels $\left\{C_{1},\cdots ,C_{J}\right\}$. The values reported in Fig. 6 reflect the mean of these average perplexity scores aggregated over all 5,000 out-of-sample iterations.

#### Prospect theory.

To benchmark our reason-based models, we implemented a hierarchical version of prospect theory (PT). Consistent with recent large-scale findings (13), we used the original, noncumulative version. The subjective value V(A) of a lottery A with outcomes $x_{i}$ and probabilities $p_{i}$ is

$$V\left(A\right)=\sum_{i=1}^{n}w\left(p_{i}\right)\cdot v\left(x_{i}\right),$$

where v(·) is the value function and w(·) is the probability weighting function. The value function was defined as

$$v\left(x\right)=\left\{\begin{array}{cc} \text{sign}\left(x\right)\cdot {\left|x\right|}^{\alpha } & \text{if gain- or loss-only problems},\\ -\lambda \cdot {\left|x\right|}^{\alpha } & \text{if}x<0\;\text{in mixed-domain problems}. \end{array}\right.$$

The probability weighting function used the one-parameter Prelec form (57):

$$w\left(p\right)=\;\exp\left(-{\left(-\ln p\right)}^{\gamma }\right).$$

The choice probability was determined by a logistic-softmax function:

$$P\left(A\right)=\frac{1}{1+\exp\left(-\phi \cdot (V(A)-V(B))\right)},$$

where ϕ is a sensitivity parameter. We estimated the model using a Bayesian hierarchical framework in Stan, with flat priors, obtaining population and individual-level estimates for α, λ, γ, and ϕ. Out-of-sample predictive accuracy (Fig. 6) was estimated using Pareto-smoothed importance sampling leave-one-out cross-validation (PSIS-LOO) via the loo package in R (58).

The median posterior parameter estimates (with 95% Bayesian CI; BCI) were α=0.34, 95% BCI: [0.23, 0.53]; λ=1.68, 95% BCI: [0.98, 2.86]; γ=0.73, 95% BCI: [0.57, 0.88]; ϕ=0.41, 95% BCI: [0.14, 0.74].

### Clustering.

To explore variability in reasoning patterns, we applied a dimensionality reduction and clustering pipeline to the reason-by-individual and reason-by-problem matrices. First, we used PaCMAP (45) to embed the high-dimensional matrices into a two-dimensional space. Second, we performed hierarchical clustering on the resulting embeddings using Ward’s linkage criterion.

Consistent with the analysis in the main text and our desire for a parsimonious description, we extracted four clusters for participants and three for choice problems. These cluster assignments were used for visualization (Fig. 5) and as a conditioning variable in the out-of-sample prediction analysis (Fig. 6). This analysis was exploratory and intended to illustrate the structure present in the reason distributions.

## Supplementary Material

Appendix 01 (PDF)

## Data Availability

The behavioral choice data, free-text reports, demographic information, the LLM outputs, and the code allowing to reproduce all reported results are available in the public GitHub repository (https://github.com/kfulawka/llm-decision-reasons) (59).
