# Supplementary material for: Large language models accurately identify decision reasons in verbal reports
Source: Proc Natl Acad Sci U S A. 2026 Jun 30;123(27):e2526798123. doi: 10.1073/pnas.2526798123 (PMC13342909; doi:10.1073/pnas.2526798123)
Supplement: Supplementary file 1 — Appendix 01 (PDF) [file pnas.2526798123.sapp.pdf]

Supporting Information for

Large language models accurately identify decision reasons in verbal reports

Kamil Fuławka, Ralph Hertwig and Dirk U. Wulff (complete author list)

Kamil Fuławka
E-mail: kamil.fulawka@tu-dresden.de

This PDF file includes:

Supporting text
Figs. S1 to S3
Tables S1 to S5

Supporting Information Text

Materials

LLM propmts. Figure S1 and figure S2 provide the exact text of the zero-shot prompts used in the LLM-based analyses.

Decision reasons. Table S1 presents the full list of verbal representations of decision reasons used in the study. The formal representations, in a form of computer code, can be found in the public GitHub repository.

Choice problems. Table S2 presents the full list of choice problems between used in the study. Each choice problem consisted of two monetary lotteries, A and B. Each lottery consisted of up to three outcomes and their corresponding probabilities.

Results

Figure S3 shows additional results of reason–choice alignment for LLMs of different sizes. Table S3 reports the variance decomposition of LLM-identified decision reasons across participants and choice problems, quantifying the relative contributions of problem structure, individual differences, and their interactions. Table S4 summarizes posterior differences in out-of-sample predictive accuracy between models based on verbal and formal decision reasons across different conditioning schemes. Table S5 reports posterior differences in perplexity. Tables S4 and S5 together indicate that verbal-reason models achieve comparable or better predictive performance while relying on more parsimonious reason distributions. All models were estimated in `R` using `brms`. The full analysis code, including model specifications and data processing pipelines, is available in the project's GitHub repository and can be used to reproduce all reported results.

Predicting choice from verbal reports and identified decision reasons. As an additional test of the predictive information contained in verbal reports, we evaluated whether participants' choices could be predicted directly from trial-level representations of their reports. First, we embedded each of the 1,720 raw verbal reports using Qwen3-Embedding-8B. Each report was formatted with the instruction: "Given an individual's verbal report about a choice between monetary lotteries, retrieve the relevant information on decision reasons," followed by the report text, and embeddings were generated with vLLM and saved together with participant, problem, and report metadata. The embedding model produces 4,096-dimensional embeddings; however, because it is based on a Matryoshka representation design, lower-dimensional prefixes of the embedding vector can be used for downstream prediction. Accordingly, we used the first 1,024 embedding dimensions in the present analysis.

We then fit ridge-penalized logistic regression models to predict the binary choice outcome from different trial-level report representations. Predictive performance was evaluated out-of-sample using five-fold cross-validation, with folds assigned within participants, repeated 100 times. The regression from the embedding model achieved 84% out-of-sample accuracy. In contrast, a regression based on the interpretable LLM-derived reason representations achieved substantially higher accuracy: a binary vector indicating which of the 47 candidate reasons were identified with confidence equal to or above 80 yielded 93.4% accuracy, whereas using the LLM confidence scores for all reasons yielded 95.4% accuracy.

These results show that the LLM-extracted decision reasons preserve nearly all choice-relevant information contained in the reports while reducing them to an interpretable set of decision-process features. At the same time, all trial-wise prediction models require a verbal report for the specific choice being predicted. By contrast, the reason-profile models reported in the main text extract reusable reason distributions, including problem-specific profiles, that can be used to predict choices even when no new verbal report is available for a given trial and shed light on the components of the underlying decision process.

```
SYSTEM MESSAGE
You are a decision analyst who accurately applies decision reasons to choice problems and identifies which lottery the decision
reason prefers or whether the decision reason is indifferent between the lotteries.

Available information --
A choice problem poses a choice between two lotteries, A and B, offering different monetary outcomes with different
probabilities.
A decision reason specifies a rule to decide which of the two lotteries is preferred by the reason.  The decision reason
prefers A or B or is indifferent between the lotteries.

Task description --
Your task is to identify the lottery preferred by the decision reason by applying the reason to the choice problem step-by-step.
Here are the steps:

Step 1:  Assess if the decision reason can be applied to the choice problem in a strict manner.  This requires evaluating
whether the relevant information can be derived from the lotteries' outcomes and probabilities.  If the relevant information
cannot be derived, you should be indifferent between the lotteries and return INDIFFERENT as the final answer.  Otherwise,
summarize the information about the lotteries relevant to the decision reason and proceed to Step 2.

Step 2:  Apply the decision reason to the choice problem by strictly following the decision reason's rule.  Use the relevant
information summarized in Step 1.  If the decision reason prefers both lotteries equally, return as the final answer
INDIFFERENT. Otherwise, if the decision reason prefers one lottery, return as the final answer A for lottery A or B for lottery
B.

Output structure - use the following template:
STEPS: <describe each reasoning step>
FINAL ANSWER: <provide final answer>

--
USER MESSAGE (example instance)
choice problem:
Lottery A:
2500 Euros with 33% probability
0 Euros with 67% probability

Lottery B:
2400 Euros with 34% probability
0 Euros with 66% probability

Decision reason:
The reason considers the maximum outcome of each lottery.  The reason prefers the lottery with the more favorable maximum
outcome.
```

Fig. S1. Full prompt used in the task of validating LLMs' understanding of choice problems and reasons.

```
SYSTEM MESSAGE
You are a decision analyst specializing in evaluating decision-making processes.
Your task is to assess whether a specific decision reason is present in a verbal report of an individual who have made a choice
between two monetary lotteries.

Available information --
A choice problem poses a choice between two lotteries, A and B, offering different monetary outcomes with different
probabilities.

A decision reason specifies a rule to decide which of the two lotteries is preferred by the reason.  The decision reason
prefers A or B or is indifferent between the lotteries.

A verbal report written by an individual describes, in retrospect, the individual's deliberation process used to choose one of
the lotteries of the choice problem.

Task description --
Your task is to assess, based on the verbal report, whether the individual used the reason to make the decision.  Conduct the
assessment step-by-step.  Here are the steps:

Step 1:  Assess if the decision reason can be applied to the choice problem.  Evaluate whether the information relevant to the
decision reason can be derived from the lotteries' outcomes and probabilities and summarize this information.
Proceed to Step 2.

Step 2:  Assess the verbal report.  First, evaluate and summarize information considered by the individual.  Second, evaluate
and summarize the individual's justification for the choice.  Focus on the described deliberation process and ignore
information about the individual's final choice.  Proceed to Step 3.

Step 3:  Assess whether the decision reason was used by the individual.  First, compare the information relevant to the
decision reason with that considered by the individual.  Second, compare the decision reason's rule to the individual's
justification for the choice.  Based on these two comparisons, assess whether the individual used the decision reason to make
the decision.
Indicate your final assessment on a scale between 0 and 100.
Interpret the endpoints and the midpoint in the following way:
- 0:  Certain that the decision reason was not used by the individual.
- 50:  Uncertain whether the decision reason was used or not used by the individual.
- 100:  Certain that the decision reason was used by the individual.

Output structure--use the following template:
STEPS: <describe each reasoning step>
FINAL ASSESSMENT: <provide final numerical assessment>

--
USER MESSAGE
As in Box 1, extended with a verbal report text for a given trial.
```

Fig. S2. Full prompt used in the task of identifying decision reasons in verbal reports with Lllama-3.3-70b.

Table S1. Decision reasons and their descriptions.

Decision reason name	Decision reason description
minimum outcome	The reason considers the minimum outcome of each lottery. The reason prefers the lottery with the more favorable minimum outcome.
maximum outcome	The reason considers the maximum outcome of each lottery. The reason prefers the lottery with the more favorable maximum outcome.
higher maximum probability	The reason considers the maximum probability of each lottery. The reason prefers the lottery with the higher value of maximum probability.
lower maximum probability	The reason considers the maximum probability of each lottery. The reason prefers the lottery with the lower value of maximum probability.
higher minimum probability	The reason considers the minimum probability of each lottery. The reason prefers the lottery with the higher value of minimum probability.
lower minimum probability	The reason considers the minimum probability of each lottery. The reason prefers the lottery with the lower value of minimum probability.
higher minimum outcome probability	The reason considers the probability of the minimum outcome of each lottery. The reason prefers the lottery with the higher probability of the minimum outcome.
lower minimum outcome probability	The reason considers the probability of the minimum outcome of each lottery. The reason prefers the lottery with the lower probability of the minimum outcome.
higher maximum outcome probability	The reason considers the probability of the maximum outcome of each lottery. The reason prefers the lottery with the higher probability of the maximum outcome.
lower maximum outcome probability	The reason considers the probability of the maximum outcome of each lottery. The reason prefers the lottery with the lower probability of the maximum outcome.
zero outcome presence	The reason considers the presence of a zero outcome in each lottery. The reason prefers the lottery with a zero outcome.
zero outcome absence	The reason considers the presence of a zero outcome in each lottery. The reason prefers the lottery without a zero outcome.
least likely outcome	The reason considers the least probable outcomes, that is the most favorable outcome with the lowest (minimum) probability of each lottery. The reason prefers the lottery with the more favorable least probable outcome.
most likely outcome	The reason considers the most probable outcomes, that is the most favorable outcome with the highest (maximum) probability of each lottery. The reason prefers the lottery with the more favorable most probable outcome.
higher zero outcome probability	The reason considers the probability of a zero outcome of each lottery. The reason prefers the lottery with the higher probability of the zero outcome.
lower zero outcome probability	The reason considers the probability of a zero outcome of each lottery. The reason prefers the lottery with the lower probability of the zero outcome.
sure outcome presence	The reason considers the presence of a sure outcome in each lottery. The reason prefers the lottery with a sure outcome.
sure outcome absence	The reason considers the presence of a sure outcome in each lottery. The reason prefers the lottery without a sure outcome.
sum of outcomes	The reason considers the sum of all outcomes of each lottery, which is calculated by adding the outcomes of each lottery independent of their probabilities. The reason prefers the lottery with the higher outcome sum.
large outcome range	The reason considers the difference between the maximum and minimum outcome of each lottery. The reason prefers the lottery with the larger difference between the minimum and the maximum outcome.
small outcome range	The reason considers the difference between the maximum and minimum outcome of each lottery. The reason prefers the lottery with the smaller difference between the minimum and the maximum outcome.
large probability range	The reason considers the difference between the minimum and maximum probability of each lottery. The reason prefers the lottery with the larger difference between the minimum and maximum probability.
small probability range	The reason considers the difference between the minimum and maximum probability of each lottery. The reason prefers the lottery with the smaller difference between the minimum and maximum probability.
large variance	The reason considers the variance of each lottery. The reason prefers the lottery with the larger variance.
small variance	The reason considers the variance of each lottery. The reason prefers the lottery with the smaller variance.

Continued on next page

Table S1 – continued from previous page

Decision reason name	Decision reason description
mean outcome	The reason considers the mean of all outcomes of each lottery, which is calculated by adding the outcomes of each lottery independent of their probabilities and dividing by the number of outcomes. The reason prefers the lottery with the higher mean outcome.
expected value	The reason considers the expected value of each lottery. The reason prefers the lottery with the higher expected value.
outcome sensitivity	The reason considers the outcomes of each lottery. The differences between the outcomes of the lotteries are considered important. The reason prefers the lottery with the more favorable maximum outcome, or if the maximum outcomes are identical, the more favorable minimum outcome.
outcome neglect	The reason considers the outcomes of each lottery. The differences between the outcomes of the lotteries are considered negligible. The reason prefers the lottery with the higher probability of obtaining a favorable outcome.
probability neglect	The reason considers the probabilities of each lottery. The differences between the probabilities of the lotteries are considered negligible. The reason prefers the lottery with the more favorable maximum outcome.
probability sensitivity	The reason considers the probabilities of each lottery. The differences between the probabilities of the lotteries are considered important. The reason prefers the lottery with higher probability of obtaining a favorable outcome.
small probability overweighting	The reason considers the small probability outcomes of each lottery. Outcomes with a probability of less than 20% receive a weight increased by 10 percentage points than their objective probability. The reason prefers the lottery with the more favorable sum of outcomes weighted by their probabilities.
loss aversion	The reason considers the outcomes of each lottery. Losses are considered more important than gains. The reason prefers the lottery with the more favorable maximum loss.
loss avoidance	The reason considers the probabilities of losses of each lottery. The reason prefers the lottery with the overall lower probability of loss.
reference point	The reason considers the outcomes of each lottery and a reference value. The reference value is the value of a sure outcome with 100% probability if the decision problem contains a sure outcome or otherwise zero. The differences between the outcomes and the reference value are considered important. The reason prefers the lottery with the more favorable mean difference considering the differences' probabilities.
segregation	The reason considers the outcomes of each lottery. The outcome with the minimum absolute value is considered a sure outcome. The differences between the sure outcome and the remaining outcomes are considered important. The reason prefers the lottery with the more favorable sum of the sure outcome and the differences weighted by their probabilities.
maximum outcome expectation	The reason considers the products of the maximum outcome and its probability of each lottery. The reason prefers the lottery with the higher product.
minimum outcome expectation	The reason considers the products of the minimum outcome and its probability of each lottery. The reason prefers the lottery with the higher product.
importance sampling	The reason considers the maximum magnitude outcome of each lottery. The maximum magnitude outcome is the outcome with the highest absolute value. The reason prefers the lottery with the more favorable maximum magnitude outcome weighted by its probability.
regret	The reason considers the outcomes of each lottery. The sum of all pairwise differences of outcomes between the lotteries is considered important. The reason prefers the lottery with the more favorable sum of outcome differences.
disappointment	The reason considers the outcomes of each lottery. The differences between maximum and minimum outcome within a lottery are considered important. The reason prefers the lottery with a smaller difference between the maximum and the minimum outcomes.
left skewness	The reason considers the skewness of each lottery. A lottery is left-skewed if a greater portion of probability is assigned to favorable as opposed to unfavorable outcomes. The reason prefers the lottery that is more left-skewed.
right skewness	The reason considers the skewness of each lottery. A lottery is right-skewed if a greater portion of probability is assigned to unfavorable as opposed to favorable outcomes. The reason prefers the lottery that is more right-skewed.
outcomes better than average	The reason considers the number of outcomes higher than the average outcome. The average outcome is calculated by taking the mean of outcomes from both lotteries, without weighting by their probabilities. The reason prefers the lottery with the larger number of outcomes higher than the average outcome.

Continued on next page

Table S1 – continued from previous page

Decision reason name	Decision reason description
consequence count	The reason considers the comparisons of maximum and minimum outcomes between lotteries. The reason prefers the lottery with a higher number of favorable comparisons.
mean of probable outcomes	The reason considers the probable outcomes of each lottery. An outcome is categorized as probable if its probability is higher than one divided by the number of lottery outcomes. The reason prefers the lottery with a higher average of probable outcomes.
aspiration level	The reason considers the aspiration level. The aspiration level is calculated as the mean of all outcomes in the decision problem while ignoring their probabilities. The differences between the outcomes and the aspiration level are considered important. The reason prefers the lottery with the higher probability of positive differences.

Table S2. The monetary choice problems used in the study.

ID	A outcomes (€)			A probs (%)			B outcomes (€)			B probs (%)			Domain	Type
1	2500	2400	0	33	66	1	2400	0	0	100	0	0	gain	risk-safe
2	2500	0	0	33	67	0	2400	0	0	34	66	0	gain	risk-risk
3	4000	0	0	80	20	0	3000	0	0	100	0	0	gain	risk-safe
4	4000	0	0	20	80	0	3000	0	0	25	75	0	gain	risk-risk
5	6000	0	0	45	55	0	3000	0	0	90	10	0	gain	risk-risk
6	6000	0	0	0.1	99.9	0	3000	0	0	0.2	99.8	0	gain	risk-risk
7	-4000	0	0	80	20	0	-3000	0	0	100	0	0	loss	risk-safe
8	-4000	0	0	20	80	0	-3000	0	0	25	75	0	loss	risk-risk
9	-6000	0	0	45	55	0	-3000	0	0	90	10	0	loss	risk-risk
10	-6000	0	0	0.1	99.9	0	-3000	0	0	0.2	99.8	0	loss	risk-risk
11	1000	0	0	50	50	0	500	0	0	100	0	0	gain	risk-safe
12	-1000	0	0	50	50	0	-500	0	0	100	0	0	loss	risk-safe
13	6000	0	0	25	75	0	4000	2000	0	25	25	50	gain	risk-risk
14	-6000	0	0	25	75	0	-4000	-2000	0	25	25	50	loss	risk-risk
15	5000	0	0	0.1	99.9	0	5	0	0	100	0	0	gain	risk-safe
16	-5000	0	0	0.1	99.9	0	-5	0	0	100	0	0	loss	risk-safe
17	3000	-3000	0	50	50	0	1500	0	0	100	0	0	mixed	risk-safe
18	3000	-3000	0	50	50	0	2000	0	0	100	0	0	mixed	risk-safe
19	3000	-3000	0	50	50	0	1000	0	0	100	0	0	mixed	risk-safe
20	6000	-6000	0	50	50	0	0	0	0	100	0	0	mixed	risk-safe

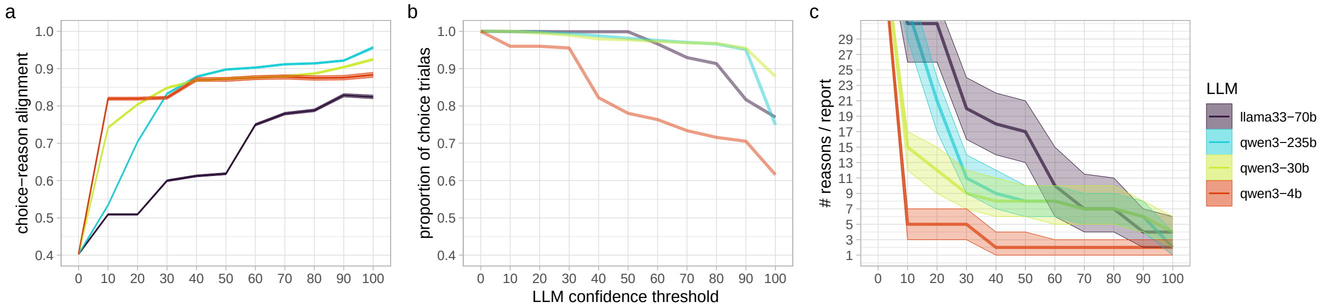

Fig. S3. Choice–reason alignment as a function of the LLM confidence threshold, shown separately for models of different sizes. For each large language model (LLM), decision reasons were counted as identified when the model's confidence assessment was at or above the indicated threshold (0–100). Panel (a) shows the mean alignment between identified reasons and observed choices across trials, where alignment indicates that an individual reason implied the participant's actual choice. Shaded ribbons indicate ± 1 standard error around the mean. Panel (b) shows the proportion of choice trials retained at each threshold, that is, trials for which at least one reason met the threshold criterion. Panel (c) shows the median number of identified reasons per verbal report, with shaded ribbons indicating the interquartile range. Importantly, the alignment measure shown here in panel (a) differs from the majority-rule alignment reported in the main text (Fig. 3b): here, alignment is computed at the level of individual reasons, whereas the main text evaluates alignment after aggregating identified reasons within each trial. Across models, increasing the confidence threshold generally improved reason-level alignment, but reduced the proportion of retained trials and the number of identified reasons per report. Notably, larger models exhibited a more favorable trade-off, with high-confidence thresholds yielding stronger gains in alignment alongside comparatively smaller losses in coverage, suggesting more informative and better-calibrated use of confidence.

Table S3. Variance decomposition of LLM-identified decision reasons.

	Variance			Proportion of variance explained		
Component	Median	2.5%	97.5%	Median	2.5%	97.5%
Problem	0.207	0.072	0.529	0.019	0.007	0.048
Reason	3.213	2.174	5.131	0.298	0.222	0.406
Reason × Problem	3.578	3.143	4.068	0.331	0.278	0.377
Reason × Participant	0.315	0.271	0.364	0.029	0.023	0.035
Participant	0.155	0.109	0.227	0.014	0.010	0.021

Note. Variance components estimated from a Bayesian logistic hierarchical regression (`brms`) with the formula `reason_present ~ 1 + (1|reason) + (1|participant) + (1|choice_problem) + (1|reason:participant) + (1|reason:choice_problem)`. The binary outcome indicates whether a decision reason was identified in a given trial (LLM confidence ≥ 80). Values are posterior medians and 95% credible intervals. Proportions reflect each component's share of total latent variance, including the logistic residual variance $\pi^2/3$.

Table S4. Posterior differences in out-of-sample accuracy between verbal and formal reason models.

Conditioning scheme	Posterior $p(\beta > 0)$	Median	2.5%	97.5%
Marginal	0.812	0.027	-0.032	0.088
Participant	0.965	0.054	-0.004	0.112
Problem	0.887	0.024	-0.015	0.061
Problem class	0.855	0.044	-0.043	0.122

Note. Reported values summarize the posterior for the fixed effect of reason type (`set`) from separate Bayesian beta regressions fit with `brms` for each conditioning scheme. Accuracy was modeled as `accuracy` $\sim$ `set + (1|participant)`, with a beta likelihood and cauchit link. Sum contrasts were used for `set`, such that positive coefficients indicate higher predictive accuracy for verbal than for formal reason models. Posterior medians and 95% credible intervals are reported on the cauchit (link) scale. The table also reports the posterior probability that the effect exceeds zero.

Table S5. Posterior differences in perplexity between verbal and formal reason models.

Conditioning scheme	Posterior $p(\Delta < 0)$	Median	2.5%	97.5%
Marginal	0.98	-12.76	-22.06	-1.05
Participant	1.00	-17.03	-18.49	-14.88
Problem	1.00	-9.87	-15.28	-7.05
Problem class	1.00	-13.69	-17.58	-9.14
Overall	1.00	-13.43	-16.16	-10.14

Note. Differences in perplexity (verbal minus formal reason models) were estimated using a Bayesian regression model fit with `brms`: `perplexity` $\sim$ `set` $\times$ `cond_g, sigma` $\sim$ `set, alpha` $\sim$ `set`, with a skew-normal likelihood. This specification allows both the variance (σ) and skewness (α) of the distribution to differ between verbal and formal reason models. Posterior predictions were computed for each conditioning scheme, and differences were calculated at the posterior draw level. Negative values indicate lower perplexity (i.e., more parsimonious reason distributions) for verbal reason models. The table reports the posterior probability that the difference is below zero, posterior medians, and 95% credible intervals.
